# The impact of the perception of rhythmic music on self-paced oscillatory movements

**DOI:** 10.3389/fpsyg.2014.01037

**Published:** 2014-09-16

**Authors:** Mathieu Peckel, Thierry Pozzo, Emmanuel Bigand

**Affiliations:** ^1^Laboratoire d'Etude de l'Apprentissage et du Développement, Centre National de la Recherche Scientifique, Université de BourgogneDijon, France; ^2^Unité 1093, Cognition, Action et Plasticité Sensorimotrice, Institut National de la Santé et de la Recherche MédicaleDijon, France; ^3^Institut Universitaire de France, Université de Bourgogne, UFR STAPSDijon, France; ^4^Department of Robotics, Brain and Cognitive Sciences, Istituto Italiano di TecnologiaGenoa, Italy

**Keywords:** embodied music cognition, oscillatory movements, musical tempo, motor resonance, motor constraints, musical affordance, motion capture, music perception

## Abstract

Inspired by theories of perception-action coupling and embodied music cognition, we investigated how rhythmic music perception impacts self-paced oscillatory movements. In a pilot study, we examined the kinematic parameters of self-paced oscillatory movements, walking and finger tapping using optical motion capture. In accordance with biomechanical constraints accounts of motion, we found that movements followed a hierarchical organization depending on the proximal/distal characteristic of the limb used. Based on these findings, we were interested in knowing how and when the perception of rhythmic music could resonate with the motor system in the context of these constrained oscillatory movements. In order to test this, we conducted an experiment where participants performed four different effector-specific movements (lower leg, whole arm and forearm oscillation and finger tapping) while rhythmic music was playing in the background. Musical stimuli consisted of computer-generated MIDI musical pieces with a 4/4 metrical structure. The musical tempo of each song increased from 60 BPM to 120 BPM by 6 BPM increments. A specific tempo was maintained for 20 s before a 2 s transition to the higher tempo. The task of the participant was to maintain a comfortable pace for the four movements (self-paced) while not paying attention to the music. No instruction on whether to synchronize with the music was given. Results showed that participants were distinctively influenced by the background music depending on the movement used with the tapping task being consistently the most influenced. Furthermore, eight strategies put in place by participants to cope with the task were unveiled. Despite not instructed to do so, participants also occasionally synchronized with music. Results are discussed in terms of the link between perception and action (i.e., motor/perceptual resonance). In general, our results give support to the notion that rhythmic music is processed in a motoric fashion.

## Introduction

The notion that perception is linked to action is not new. William James famously claimed that “*every representation of a movement awakens in some degree the actual movement which is its object*” in his ideomotor theory of action (James, 1890). In more recent years, McGurk and MacDonald (1976) notably demonstrated that the auditory perception of spoken sounds interacted with the perception of the facial movements used to produce syllables (i.e., the McGurk effect). Liberman and Mattingly (1985) further theorized about this in their motor theory of speech perception, postulating that hearing speech automatically activated the corresponding motor commands necessary to produce the sounds, ultimately facilitating comprehension in the listener. A few years later, Jeannerod (1994), Decety (1996), as well as Berthoz (1997) suggested that there was a motor aspect in the perceptual system. They suggested that the perception or imagery of an action led to the internal simulation of that action at a neural level. Insights into the neural substrate of this motor simulation in perception came with the discovery of the mirror neuron system in chimpanzees (Gallese et al., 1996; Rizzolatti et al., 1996; Rizzolatti and Craighero, 2004 for a review). These mirror neurons were described as a specific type of neurons that activated during both action observation and performed actions, directly coupling perception to action. They were first studied in the visuomotor domain where the observation of an action led to the same activation as the actual action being performed by the observer. Much like speech in humans, the same sensorimotor interactions were also observed in the audio-motor domain, where listening to the typical sound produced by a learned action activated premotor areas in monkeys (e.g., Kohler et al., 2002).

These discoveries were in contradiction with the classical cognitive theories at the time which depicted the human mind as an information processor that relied on abstract, a-modal representations of the world. This traditional perspective, despite its success, began to be criticized for neglecting the relationship between the body and its environment (e.g., Varela, 1993). In that sense, amounting experimental evidence demonstrated that bodily states, perceptual systems and actions underlie information processing (see Wilson, 2002). This new approach, referred to as “embodied cognition” emphasizes the fact that cognition is grounded in sensorimotor processes and that knowledge includes sensorimotor representations of both motor and perceptual information (e.g., Barsalou, 2008; Shapiro, 2010). Within this embodied cognition framework, Prinz (1997) as well as Hommel et al. (2001) suggested that the planning or the execution of an action and the perception of its related sensory consequences are encoded in a shared representation in the brain. As a result, whenever one of the two components is activated, both motor and sensory areas in the brain are recruited. This combination of sensory and motor representations leads to the creation of internal models relative to the relationship between the two. In that sense, these models can either contain inverse or forward components (Wolpert et al., 1995). On the one hand, inverse models (also known as *motor resonance*) refer to the way perception activates the corresponding motor commands required for such sensory state to be achieved (e.g., Jeannerod, 1994; Prinz, 1997; Rizzolatti et al., 2001). On the other hand, forward models (also known as *perceptive resonance*) refer to the way actions activate sensory states. In other words, the system predicts the sensory outcomes of the executed or planned action (Davidson and Wolpert, 2005 for a review).

In an effort to further the understanding of this relationship between action and perception, the embodied cognition theories have also been applied to music cognition which was deemed appropriate due to its close link with movement (Zatorre et al., 2007). Like Clarke (2005) suggested, music is movement in the sense that movements have to be produced in order to create music. Accordingly, theories of embodied music cognition (e.g., Leman, 2008) suggested that both movements (e.g., playing an instrument) and their perceptual consequences (e.g., tones) were encoded in a shared representation (Godøy and Leman, 2010). For example, Repp and Knoblich (2007) showed that the direction of the movement on a piano keyboard determined how an ambiguous rising/falling tone was perceived (i.e., perceptual resonance). The bias was congruent with how rising and falling tones are usually played on a piano (left to right and right to left, respectively). Moreover, Drost et al. (2005) found a motor resonance effect in guitarists when a note that was not the note beginning the piece they had to play was heard. Participants effectively took more time to start playing when the note was incongruent than when it was congruent. In line with Kohler et al.'s (2002) study, neuroimaging studies have further documented that music listening was associated with an activity in the motor areas of the brain in experts and novice alike (Haueisen and Knösche, 2001; Grahn and Brett, 2007; Lahav et al., 2007). Bangert et al. (2006) showed that the opposite was also true in the sense that playing on a muted piano keyboard also led to auditory activations in the brain. Interestingly, as Godøy et al. (2006) showed, these auditory-motor associations were also expressed through spontaneous movements in responses to music. In their experiment, participants were asked to pretend playing the piano (i.e., playing an “air instrument”) according to the piano performance they were hearing. Godøy et al. (2006) found that movements relative to synchrony with the beat and the rendering of the dynamic of the musical piece were relatively good for novices and experts alike.

In that sense, in addition to this ecological instrumental knowledge about music, research has also been devoted to understanding the link between music and movement through the spontaneous movements usually performed when listening to it (e.g. Lesaffre et al., 2008; Keller and Rieger, 2009). For example, Naveda and Leman (2010), Toiviainen et al. (2010), as well as Burger et al. (2013) suggested that music-induced movements were mainly built on the musical pulse (i.e., the beat) in expert and untrained dancers alike. Dancers would take the beat as a temporal cue and build movements around the pulse, synchronizing their movements with it (Naveda and Leman, 2010). They also suggested that the dance movements were sensitive to variations in rhythmic features (Burger et al., 2013) and that they were performed in a hierarchical fashion according to different metrical levels (Toiviainen et al., 2010). Listeners would in fact embody the different metrical levels by performing rhythmic movements with different parts of their body according to the part's motor capabilities. More precisely, lower metrical levels (i.e., slower) were associated with limbs that could only move slowly (e.g., torso) while higher metrical levels (i.e., faster) were represented with limbs that could move faster (e.g., hands, forearms).

Toiviainen et al. (2010) further suggested that the choice of certain limbs over others to move along to music might have been dictated by the integration of biomechanical motor constraints in the auditory-motor system. This idea that the motor system constrains the way we interact with music was also conveyed by Clarke (2005) and Godøy (2010) in their ecological approach to music. They suggested that listeners should be more likely to resonate in a corporeal way with music that could afford movement (i.e., motor resonance, see also Large, 2008). Additionally, Todd et al. (2007) as well as Dahl et al. (2014) have demonstrated that the individual motor constraints had an impact on how music is perceived through perceptual resonance and also on how musical preferences might be formed. The perception of the “groove” of music might also rely on a similar assessment of the motor potential of music depending on the capabilities of the listeners (Keil and Feld, 1994; Iyer, 2002). When the music “feels right” to the listeners, they might start moving different parts of their body along to music (Madison, 2006; Janata et al., 2012). These findings further suggest that music perception is really a perceptual as much as a motor activity.

On a neurobiological level, these motor constraints appear to be hardwired in the motor system itself and express themselves through optimal movements, allowing the production of motion at a minimal energetic cost (Todorov, 2004). These constraints are most noticeable in a simple system such as pendular rhythmic movements. As Holt et al. (1995) have suggested, these pendular movements are most energy efficient when they are performed at their resonant frequency, which is determined by the physical features of the pendulum (i.e., length, mass, etc. of the limb). These particular rhythmic movements are thought to be effectively tuned to their resonant frequency by neural networks known as central pattern generators (CPG) (e.g., Verdaasdonk et al., 2006). Taking into account its kinematic parameters (e.g., gravity, limb used, joint viscosity, etc.), CPGs generate alternated stimulation of antagonist muscles groups to tune a limb to its resonant frequency and produce optimal rhythmic movements (see MacKay-Lyons, 2002 for a review).

In order to better understand how music might be related to movement, researchers in embodied music cognition also studied the interaction between music and some of these constrained oscillatory movements. For example, Styns et al. (2007) and Leman et al. (2013) were interested in understanding how walking (i.e., the most common action relying on CPGs, see Miall, 2007) could be related to listening to rhythmical music. They asked participants to synchronize their walking pace to various rhythmical musical pieces and found that participants were mostly accurate in synchronizing their walking with the music through a variety of tempi. Interestingly, their synchronization with music was most accurate when its tempo was around 120 BPM (or 2 Hz) which is usually associated with the optimal walking pace (see MacDougall and Moore, 2005) as well as the most perceived and represented tempo in a very large variety of western musical pieces (over 74,000 pieces, see van Noorden and Moelants, 1999; Moelants, 2002).

In the current study, in a similar fashion, we suggested investigating the way in which music is related to movements in listeners by examining how motor resonance in the context of music perception might be mediated by motor constraints. Recently, Demos et al. (2010) investigated the impact of background music on rhythmic movements performed at a preferred tempo (self-paced). They asked participants that sat in a rocking chair to sway at their preferred rate during a memory task whilst music was played in the background. They found that listeners were sensitive to the musical beat even when they were not instructed to attend it. However, as suggested by Demos et al. (2010) themselves, their experiment could only provide data on self-paced movements related to the use of the rocking chair. Rocking chairs having a resonant frequency of their own, participants merely adapted their movements to keep the rocking chair going, compromising the very notion of “self-paced” movements.

The current study went further in investigating the impact of rhythmic music perception on cyclic movements using limbs which biomechanical constraints may differ. This allowed us to oppose the natural tendency of people to move at an optimal tempo (i.e., motor constraints) and their tendency to interact with music which might resonate with their motor system (i.e., motor resonance) in a wider range than previously studied by Demos et al. (2010). Ultimately, we were interested in documenting how individuals would, if ever, adjust their movements in the presence of a musical beat, depending on the limb used to move and the tempo of the music. In order to examine this particular issue, we conducted an experiment in two parts. The first part examined the kinematics of different self-paced oscillatory movements (i.e., expressing the motor constraints) along with walking and finger tapping. The purpose of this part was to have a better understanding of the parameters we would try to modify later. In the second part of the study, we wanted to examine the effect of music perception on self-paced oscillatory movements. Based on the assumption that different limbs are tuned to different resonant frequencies, we used rhythmic music, the tempo of which was increasing by increments. This increasing tempo would allow us to study the online influence of music on movement as well as to vary the amount of attractivity (or interference) the music would have on the movements. We could then expect that music which tempo is near the resonant frequency of a movement to interfere to a greater extent than music which tempo is more distant. Furthermore, we also wanted to investigate precise moments in the experiments; namely when music started, stopped and, if applicable, when participants' movements were synchronized with the music.

## Study one

### Introduction

The purpose of this first study was to investigate the kinematic parameters of oscillatory movements. Namely, we were most interested in the hierarchy of the different movements depending on their proximal/distal characteristics. According to the biomechanical constraints account, we should observe that the different limbs' resonant frequencies support a hierarchical organization. More precisely, if we consider a particular limb as a compound pendulum consisting of a rigid body swinging around its axis (i.e., joint), its length and mass should determine its period of oscillation. Because more distal limbs are characterized by a shorter length and lower mass than their depending proximal limb (e.g., forearm compared to whole arm), they should oscillate faster. In that sense, more distal limbs should have a higher natural frequency than more proximal limbs. Furthermore, we also predict that functionally related limbs (i.e., hip and shoulder in walking) should have similar natural frequencies because they rely on a coordinate action during walking (Wagenaar and Van Emmerik, 2000).

### Materials and methods

#### Participants

A total of 11 participants took part in this first study. Six men and five women participated in this study. Their average age was 28 year old. All the participants were either Master, PhD, or Post-doc students from the University of Burgundy.

#### Apparatus

**Figure 1:**
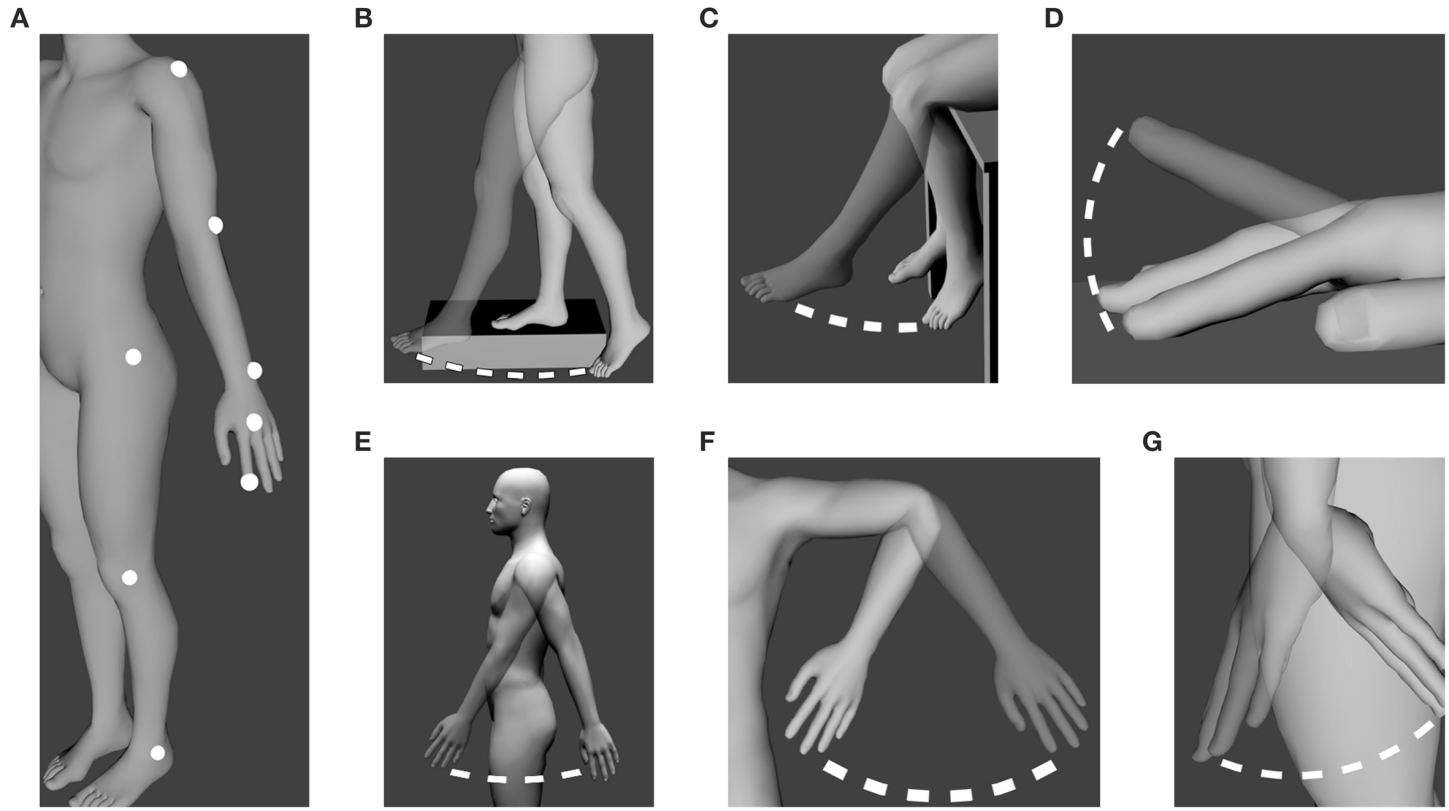
**Location of the markers and performed movements. (A)** Location of the eight markers on the participants. **(B)** Whole leg oscillation (*Hip*). **(C)** Lower leg oscillation (*Knee*). **(D)** Finger tapping (*Tapping*). **(E)** Whole arm oscillation (*Shoulder*). **(F)** Forearm oscillation (*Elbow*). **(G)** Hand oscillation (*Wrist*). All movements were performed in the sagittal plane except **(F)** which was performed in the coronal plane. The male human model is part of the open source modeling tool “Make Human” (www.makehuman.org).

The experiment took place in the purposely built Motion Capture laboratory available at the INSERM U1093 laboratory. A BTS SMART 3D Motion Capture System (BTS Bioengineering Corp., NY, USA) (sampling rate: 120 Hz) was used to capture the participants' movements. The nine cameras provided with the system were placed in a 210° semi-circle around the area where the participants had to perform their movements. Six cameras were placed about 3 m from the area while the remaining three cameras were placed 1.5 m from the participant to allow for precise measurement of small movements. Eight passive markers were placed on each participant: their location can be found in Figure 1A. Markers were put on the side that participants felt most comfortable with.

#### Procedure

Participants were required to perform six different self-paced oscillatory movements. Before each movement, participants were asked to perform a small memory test. The experimenter gave them a short story to read and asked them to recall it immediately after^1^. Participants were told they would have to perform five pendulum-like oscillatory movements as well as a tapping task. Participants were asked to use only one joint at a time. Movements were explained verbally to the participants to avoid any imitational behavior. It was explained to participants that the movements had to require the least effort possible and had to be performed in the most “natural” way possible. Movements used during the experiment can be seen on Figure 1. Movements are referred to as their corresponding joint. For example, the whole leg oscillation (Figure 1B) is referred to as *Hip* while the lower leg oscillation (Figure 1C) is referred to as *Knee*.

As seen on Figure 1, additional support was given to the participants for whole leg oscillation (Figure 1B) and lower leg oscillation (Figure 1C) for maximum stability. Participants had their arm held in the air with a soft strap to minimize fatigue for the forearm oscillations (Figure 1F). Participants tapped on a table while being seated (Figure 1D).

For each movement, participants were asked to remain as constant as possible throughout the recording. Each trial lasted just over 2 min and a half in total. The experimenter asked the participant to start the movement and not to stop before being told to. The whole recording session consisted of 12 10-s trials and started after a 1 min warm-up for each movement. Movements order and stories for the memory task were randomized for each participant. Participants were told to close their eyes to avoid any visual interference.

In a second session, participants were also required to perform a walking task. Participants had to walk at their preferred pace in a 6 m long corridor drawn on the floor for a total number of 12 passages. Participants started walking before entering the 3D working space. Their movements were recorded using the same 3D Motion Capture cameras with a different camera placement (sampling rate: 120 Hz) allowing for a bigger 3D working space.

### Results

#### Data processing

In the oscillatory movements and walking tasks, raw data consisted of 3D coordinates in the 3D working space. Using a purposely built program for 3D motion capture analysis in Matlab (Mathworks Inc, MA, USA), the signal was first filtered using a low-pass Butterworth filter with a cut-off frequency of 5 Hz. The waveforms were then converted to the frequency domain using a fast Fourier transform (FFT). The dominant peak in the resulting spectrum of amplitudes was identified using a peak finding function in Matlab. Exploitable data thus consisted of the average frequency (Hz) of the 12 trials for each movement and each participant. It is noteworthy that not all markers' data were used because some markers were not relevant to analyze a limb's resonant frequency. For all the lower limb movements and the walking task (Figures 1B,C) the ankle marker was used to compute the oscillation frequency. For all the upper limb movements (Figures 1D–G) the finger marker was used to compute the oscillation frequency. In the walking task, for comparison purposes, an oscillation was deemed equivalent to the time between one foot touching the ground and the same foot touching the ground on the next step. This time corresponds to a full oscillation of the whole leg around the Hip joint. Because this oscillation had to be captured while the participant was walking, its frequency was calculated using its Y-axis coordinates.

#### Comparison between oscillatory movements

**Figure 2:**
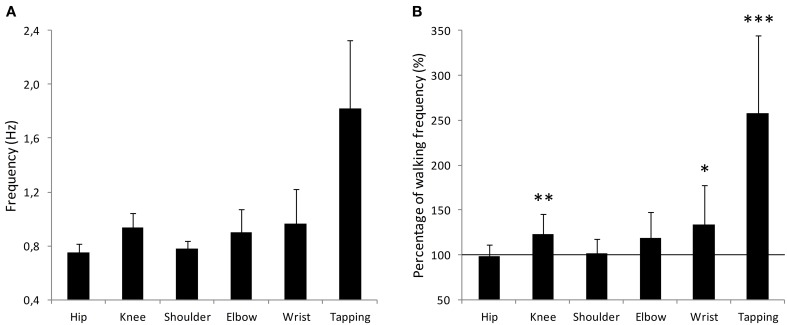
**Comparison between the six movements performed during the experiment. (A)** Mean frequency (Hz) for each movement. Movements are referred to as their corresponding joint. Error bars represent standard error. **(B)** Each movement frequency is represented relative to the mean walking frequency for each participant, corresponding to the value 100%. Error bars represent standard error. ^*^p < 0.05, ^**^p < 0.01, ^***^p < 0.001.

We were interested in examining how the different oscillatory movements were related to each other. A repeated measures ANOVA analysis revealed a main effect of the movement [*F*_(4, 36)_ = 5.19, *p* = 0.002]. A summary of the results can be found in Table 1. In order to test the hierarchy of the different oscillatory movements, planned one-tailed *t*-tests were performed. A Bonferroni comparison correction was applied to the tests in order to account for the multiple comparisons. Analyses showed that Knee movements were faster than Hip movements (*t* = 5.35, *p* < 0.001). Elbow movements were faster than Shoulder movements (*t* = 3.38, *p* = 0.017) but were not found to be faster than Wrist movements (*t* = 0.91, *p* = 0.96). Furthermore, Wrist movements were not faster than Shoulder movements (*t* = 2.31, *p* = 0.11). Finally, Hip movements were not found to be faster than Shoulder movements (*t* = 1.01, *p* = 0.83). In sum, limbs follow a hierarchical organization in the sense that distal limbs are faster than proximal limbs. However, Wrist movements were not found to be faster from more proximal arm movements (i.e., Shoulder and Elbow). It is worthy to note that the notion of “not faster than” is not interpretable as statistical analyses are meant to detect differences. The absence of statistical difference does not, under any circumstances, mean that the two values are identical. The mean frequencies of each movement can be found in Figure 2A.

**Table 1:** **Statistical differences between the five oscillatory movements (*one-tailed t-tests*)**.

	**Hip**	**Knee**	**Shoulder**	**Elbow**	**Wrist**
	**0.75 Hz (±0.06)**	**0.94 Hz (±0.11)**	**0.78 Hz (±0.06)**	**0.90 Hz (±0.17)**	**0.97 Hz (±0.26)**
Hip		5.91^***^	1.01	–	–
Shoulder				3.38^*^	2.31
Elbow					0.91

* p < 0.05,

*** *p < 0.001 (Bonferroni corrected)*.

The finger tapping task yielded the highest frequency (1.82 Hz ± 0.50) but does not qualify as a pendulum movement and thus was not comparable with the others. Nevertheless, it is interesting to note that finger tapping frequency was close to the 2 Hz usually found to be the preferred tapping rate (e.g., Fraisse, 1982; Moelants, 2002).

#### Comparison between walking frequency and other movements

The next step in our investigation was to compare the resonant frequencies of the participants' limbs to their walking frequency. For this purpose, for each participant, their mean walking frequency was given the value 100% and compared to all other movements.

All values (%) were compared to the 100 standard, representing the walking frequency. Statistical analyses revealed that Hip (mean = 98.1, *t* = −0.51, *p* = 0.620), Shoulder (mean = 101.8, *t* = 0.38, *p* = 0.712) and Elbow (mean = 118.7, *t* = 2.14, *p* = 0.057) were not different from the walking frequency (100%). The other three movements were statistically different from 100 (Knee, *t* = 3.42, *p* = 0.006; Wrist, *t* = 2.61, *p* = 0.025; Finger, *t* = 6.01, *p* < 0.001). The comparison between individual walking pace and limb movements are presented in Figure 2B. These results suggest that the Hip, Shoulder, and Elbow joints might be functionally related to walking as their natural frequencies in a self-paced pendulum-like oscillatory task are similar to the walking frequency. Once again, it is worthy to note that while statistical analyses did not reveal a statistical difference, it does not mean they are identical.

### Discussion

This first experiment investigated the relationship between different self-paced oscillatory movements performed with different limbs. Not surprisingly, results showed that the hierarchy in the natural frequency of limbs is in accordance with biomechanical constraints accounts; namely, bigger limbs oscillate more slowly than smaller limbs. In the same sense, these results also show that the proximal limbs (Hip and Shoulder) oscillate slower than their corresponding distal limbs (Elbow and Knee).

Interestingly, results also suggested that both proximal joints (Hip and Shoulder) oscillated at a similar frequency. This is particularly remarkable because the whole leg and the whole arm are different in terms of biomechanical characteristics. This would suggest that the constraints dictating the optimal frequency might not exclusively be based on the physical properties of the limb. Finding the same oscillation frequencies make sense when they are compared with the walking frequency of participants (Figure 2B). These results suggest that the constraints applied to both whole arm and leg might not be exclusively mechanical but also functional. This is in line with the results found by MacDougall and Moore (2005) who demonstrated that the mechanical properties of the body had no influence on the frequency at which walking was tuned. Because the arm and leg synchronize during walking, these two body parts might be hard-wired to function similarly even outside the context of walking.

## Study two

### Introduction

As we suggested previously, we were interested in examining how movements that were typically tuned to optimal frequencies (i.e., oscillatory movements) could spontaneously interact with music. We proposed that the previously documented motor resonance associated with music perception might interact with motor constraints. In other words, we wanted to investigate how and when music perception could resonate with the motor system in the context of these constrained oscillatory movements. In this sense, we opposed the tendency of people to move at an optimal frequency (motor constraints) and the tendency of people to move along to music (motor resonance). Furthermore, we examined this in an online and continuous fashion with music the tempo of which was increasing in order to vary the affordance of music.

Accordingly, in Study One, we found that different limbs have different natural resonant frequencies. Limbs were organized according to their proximal/distal characteristic and followed biomechanical constraints accounts. Moreover, our data suggested that Hip and Shoulder movements were not different from each other due to their functional interaction in walking.

Based on these results, the main hypothesis of this second study was that different limbs should be distinctively influenced by the perception of music. Namely, the motor resonance associated with music perception should mediated by difference motor constraints. This influence should present itself when music is first presented, when it stops and also during the whole song mainly in the form of an attraction to the musical pulse. Moreover, based on the assumption that the musical groove relies on a match between motor capabilities and relevant musical features, we should observe synchronization with music only when that match is perceived and it should be associated with pleasant feelings; when the music feels right, participants should tune in to it. In the same way, according to musical affordances accounts, participants should be more attracted to musical pulses on which they can move (i.e., musical affordance).

### Methods

#### Participants

In total, 15 new participants (i.e., different from Study One) took part. Nine women and six men participated in this second study. Their average age was 27 years old. All the participants were either Master, PhD, or Post-doc students from the University of Burgundy. Five participants reported having played (or playing) instrumental music with an average of 9.8 years of practice (min 3, max 20).

#### Apparatus

For this second study, a Vicon 3D Motion Capture system (Vicon Motion Systems, UK) was used (sampling rate: 100 Hz). Seven cameras were placed in a semi-circle around the 3D working area. One camera was placed closer to the working area in order to improve small movement measurement accuracy. The passive markers were positioned identically to the first study (Figure 1).

#### Material

Musical material consisted of six different songs purposely generated with the software Garage Band (Apple Inc., CA, USA) using MIDI loops. MIDI loops were chosen because their tempo could be changed without provoking too much distortion. The exact composition of each song is given in the Appendix section^2^. Every song was composed according to a 4/4 metric and started at a reference tempo (set in the software) of 60 BPM. The tempo increased incrementally by 6 BPM steps every 22 s. A particular tempo was maintained for 20 s and the transitions between two tempi lasted 2 s. In the end, participants heard 11 levels of tempo from 60 to 120 BPM. The total length of each song was 240 s (20 s ^*^ 11 levels + 2 s ^*^ 10 transitions). Each song was different in genre and instruments. A 1.5 s fade-in/fade-out at the beginning and ending of each song was added in order to avoid startling the participants. Music was delivered through a headset at a fixed volume for every participant.

#### Procedure

The experiment was presented as a study on relaxation through the means of self-paced oscillatory movements. Participants were told they would have to perform movements while listening to music. Participants were told the music would be increasing in tempo but should not be given attention. The participant's task was to maintain pleasant/natural/effortless movements throughout the experiment. The experimenter emphasized on the self-paced aspect of these movements.

Only four movements from the previous experiment were used. Given that whole leg and whole arm movements did not seem different in the previous experiment, only the whole arm movement was used. Moreover, the whole leg oscillation was not deemed appropriate to move along to music because it was too different from the music-induced movements involving the hip joint studied by Toiviainen et al. (2010). Namely, they found that hip movements in the context of moving along to music consisted of swaying movements with both legs supporting the whole body while our whole leg oscillatory movement was performed on one leg with the other one supporting the whole body. The wrist oscillatory movement was also dismissed as it was regarded as irrelevant to movements performed spontaneously with music. The remaining movements were thus: lower leg, whole arm, and forearm oscillations (Figures 1C,E,F, respectively) and the tapping task (Figure 1D).

Before performing each movement, participants were given at least 1 min to warm up their muscles and familiarize with the movement. When participants felt ready, the motion capture started. The whole session for one movement lasted 6 min (warming up not included). Participants started by performing the movement in complete silence for 1 min for Baseline purposes. After this 1 min Baseline, the music started and lasted for 4 min (240 s). When music stopped, participants had to continue performing the movement for one additional minute (a period referred to as “Silence”). Movements and songs were randomized for each participant. Participants were told to close their eyes to avoid visual interference.

After each movement, participants were asked to fill in a questionnaire about their subjective rating of different moments in the recording session. Participants were asked to indicate on a timeline representing the recording session when the experiment was “Hard/Unpleasant” and when it was “Easy/Pleasant.” The timeline was divided into 7 epochs: 1 epoch for the Baseline segment, 5 epochs during which music was present and 1 epoch for the Silence segment. This segmentation was used to simplify the rating. Participants were then asked to verbalize the reason why it was pleasant or unpleasant.

After having performed all the movements, participants were told to walk for 1 min in a large circle inside the Motion Capture laboratory in complete silence. As in Study One, participants started walking before their movements were recorded to ensure it was not measured from a still start.

### Results

#### Data processing

Raw data consisted of 3D (X, Y, Z) coordinates in space of all markers. Another Matlab program was purposely created to process the 3D Motion Capture data. Raw data was filtered using a one-dimensional order-7 median filter^3^. In order to compute the movement “tempo^4^,” the signal was first de-trended (i.e., centered around a 0 mean) to account for the eventual displacement of the participant inside the working area. We then extracted the onsets (in seconds) of the coordinates of all peaks along the movement axis on the same side (i.e., a full cycle, back, and forth) using a peak finding function in Matlab. The tempo was then calculated by dividing 60 by the average inter-onset interval (IOI) for a whole 20 s segment. Transitions between tempi were not taken into account.

In total, the recording was divided into 13 segments. The first segment, referred to as “Baseline” corresponded to the first minute of silence during which participants performed the movement without any music. The 11 next segments were the segments when music was present (e.g., segment 2 corresponds to 60 BPM music while segment 12 corresponds to 120 BPM music). The thirteenth segment, referred to as “Silence” corresponds to the last minute of silence at the end of the experiment.

In this second study, no motion capture data was collected for the walking task. The walking tempo of participants was measured by video analysis. Participants walked for a little more than 1 min and the walking pace was calculated by measuring the number of steps taken in 60 s. This period of 60 s started after a few steps were taken.

#### Are movements performed in silence comparable between study one and study two?

For comparison purposes, all frequencies in Study One were converted to BPM by multiplying them by 60. The normalized walking frequency in Study One was multiplied by 120 to be in accordance with the walking pace in Study Two. As we were interested in examining whether participants in study One and Two had the same baseline for each movement segment, we conducted five independent sample *t*-tests. Statistical analyses did not reveal any significant difference (Bonferroni corrected) between baselines taken from the two studies (Shoulder: *p* = 0.675; Elbow: *p* = 0.357; Knee: *p* = 0.775; Tapping: *p* = 0.889; Walking: *p* = 0.751). Like in the previous analyses, the absence of statistical difference does not mean that the values are identical. However, we can see that movements followed the same patterns as in Study One. The comparison of movements' tempi depending on the task and study is presented in Table 2.

**Table 2:** **Comparison of movements' tempo performed in silence depending on the study**.

**Movement**	**Study One (BPM)**	**Study Two (BPM)**	***t***	***p*-value**
Shoulder	46.6	47.7	0.67	0.675
Elbow	54.1	58.1	0.94	0.357
Knee	56.4	57.0	0.29	0.775
Tapping	109.4	102.4	0.14	0.889
Walking	92.9	94.0	0.32	0.751

#### Were participants influenced by the music starting, stopping or throughout the experiment?

To answer this question we focused on two particular moments in the recording session: the moment when the music started and when it stopped. In order to measure the influence of music on movements we computed three different indices: Music Starting, Music Stopping, and Baseline-Silence Difference (shortened to B-S Difference). These indices represented the variation in movement tempo between two segments of interest (SOI). In order to measure this variation, we subtracted the movement tempo observed in the first SOI from the second SOI and divided that by the tempo of the first SOI and then multiplied it by 100. In other words, it corresponds to the variation in percentage of the first SOI from the first SOI to the second SOI.

The first index, Music Starting, was meant to determine if participants were influenced when music started. We thus calculated the variation in tempo between the baseline and the first 20 s segment of music at 60 BPM (i.e., segment 2). Following the same idea, we measured the variation in tempo between the last 20 s segment of music (i.e., segment 12) and the 1 min silence period at the end of the experiment (Silence) to measure whether participants were influenced by the music stopping (i.e., Music Stopping). Finally, in order to measure the overall influence of music, we calculated the variation in tempo between Baseline and Silence segments (i.e., B-S Difference). This last index thus refers to the extent to which the music “carried” the movement of participants from the start (Baseline) to the end of the experiment (Silence). Relative and absolute values regarding these three indices are given in Table 3. Absolute variation values were calculated by taking the absolute difference in tempo between the first and second SOI instead of the relative difference. Relative values give an insight on the direction of the variation (deceleration or acceleration) while absolute values give an insight on the amplitude of the variation (regardless of the direction) of music on movements.

**Table 3:** **Mean variation (%) across participants between movement tempi for the three indices**.

**Movement**	**Relative variation (%)**			**Absolute variation (%)**		
	**Music Starting**	**Music Stopping**	**B-S Difference**	**Music Starting**	**Music Stopping**	**B-S Difference**
Shoulder	0.43	−2.09^a^	0.34	2.13^a^	4.05^a^	6.05^a^
Elbow	0.47	0.57^a^	1.61	3.01^a^	4.57^a^	9.94^a,b^
Knee	0.25	−0.29^a^	2.15^*^	0.84^a^	2.40^a,b^	2.55^a,b^
Tapping	4.57	−9.21^b^^*^	10.00	12.32^b^	10.34^b^	18.76^b^

* *Different from 0 at p < 0.05. Letters (a,b) represent groups of values that are different from each other within each column at p < 0.05*.

We first wanted to determine if the relative values were different from zero (i.e., meaning no variation). This was tested using a one-sample *t*-test with zero as the reference. Because the absolute values are not normally distributed and always have a positive mean by definition, this test was not performed on absolute values. Results showed that for Music Starting, no value was different from zero (*p* = 0.54; *p* = 0.68; *p* = 0.37; *p* = 0.41 from Shoulder to Tapping, respectively). Regarding Music Stopping, only the Tapping task yielded relative values different from zero (*t* = −3.00, *p* = 0.009). The three other values were not different from zero (*p* = 0.19; *p* = 0.69; *p* = 0.74; from Shoulder to Knee, respectively). Finally, the only significant difference in tempo found between Baseline and Silence epochs (B-S Difference) was found in the Knee movement (*t* = 3.17, *p* = 0.006). Taken together, these results suggest that during the Tapping task, participants consistently decelerated when music stopped. Furthermore, participants moved faster during the Silence epoch than during the Baseline epoch when performing the Knee movement.

We then wanted to know if these values were different from each other. To test that, we performed a repeated measure ANOVA for each index with Movement (Shoulder, Elbow, Knee, Tapping) as the only within-subject variable. We first analyzed the relative values. The Music Starting relative values were not different from each other [*F*_(3, 42)_ = 0.54; *p* = 0.65]. However, the effect of Movement was significant for the Music Stopping relative values [*F*_(3, 42)_ = 6.34; *p* = 0.001]. *Post-hoc* (Bonferroni) analyses revealed that the Tapping task was different from Shoulder (*p* = 0.04), Elbow (*p* = 0.001) and Knee (*p* = 0.005). The three other movements were not different from each other (*p* = 1). Furthermore, there was no effect of Movement for the B-S Difference index relative values [*F*_(3, 42)_ = 1.12; *p* = 0.34].

The same analyses were performed for the absolute values. The repeated-measure ANOVA revealed an effect of Movement for the Music Starting index [*F*_(3, 42)_ = 5.3; *p* = 0.003]. *Post-hoc* (Bonferroni) also revealed that the tapping task was the most influenced movement and was different from the three other movements (*p* = 0.017; *p* = 0.035; *p* = 0.005 for Shoulder, Elbow, and Knee respectively). Analyses also revealed a main effect of Movement for the Music Stopping absolute values [*F*_(3, 42)_ = 5.24; *p* = 0.003]. *Post-hoc* (Bonferroni) revealed that the Tapping task was different from the Shoulder (*p* = 0.03) and the Knee movement (*p* = 0.003). The tapping task was marginally different from the Elbow movement (*p* = 0.059). No other differences were found. Regarding the B-S Difference, analyses showed a movement effect [*F*_(3, 42)_ = 4.14; *p* = 0.01]. However, *Post-hoc* (Bonferroni) only revealed that the Tapping task was different from the Knee movement (*p* = 0.01). The Tapping task was marginally different from the Shoulder movement (*p* = 0.07) but not from the Elbow movement (*p* = 0.45). No other differences were found.

To summarize, due to a great variability across participants, it was difficult to determine if participants were influenced by music throughout the experiment or by starting and stopping. However, it appeared that the tapping task was the most influenced by music. All the other movements were mostly not different from each other and were influenced by music to a lesser extent.

#### How did participants react throughout the experiment in terms of strategies?

As suggested in the previous section regarding the influence of music, quantitatively describing the participants' behavior is difficult as they behave differently and sometimes in opposite ways. Nonetheless, some participants expressed similar strategies or behavioral patterns in all movements in reaction to music starting and its increasing tempo. These strategies are referred to as “patterns.”

The selection of the patterns was determined visually and quantitatively on the graphs representing the evolution of tempo through time for each movement and every participant. The first distinctive characteristic we looked for was whether the movement tempo was increasing, decreasing, or not changing. The patterns were then chosen based on other characteristics such as their starting point, the number of segments involved, the musical target of the movement and the quantitative difference in tempo between segments. The starting point of the pattern might refer to the Baseline or anywhere else in the session. A particular case was made for Metrical Level as a starting point (see *Metrical Change* pattern below). The musical target refers to the apparent “target” that the participant is trying to reach through the expression of a pattern. The target might concern the current metrical level, a higher or lower metrical level. A table summarizing the different criteria associated with each pattern can be found in the Appendix.

The first pattern, referred to as “*Acceleration*” corresponds to an acceleration of the movement tempo when music was present (while not being synchronized). When participants accelerated over 3 BPM in one segment or through the course of several segments (2–5), it was considered as “*Acceleration*.” This threshold of 3 BPM was chosen because it was the objective increment in tempo associated with the lowest metrical level with which participants interacted (1:2). It was decided to consider >3 BPM accelerations over several segments as Acceleration patterns because the music had to have a stimulating effect on participants to incite them to accelerate past 3 BPM. The second pattern, “*Start Adaptation*,” corresponds to the participant adapting his movement tempo when music starts. This adaptation might be a deceleration or an acceleration to get closer to a particular metrical level. This pattern only occurs after the Baseline segment. The third pattern, “*Adaptation*,” corresponds to the participant adapting his movement tempo to come closer to the musical tempo or an equivalent metrical level. In other words, the participants decelerate to get closer to the perceived musical tempo. This deceleration might either be sudden when participants are close to the musical tempo or subtle and lasting for several segments when they start far away from the musical tempo or equivalent metrical level. Contrary to the *Start Adaptation* pattern, it does not occur immediately after the Baseline and typically occurs later in the session. The fourth pattern, “*Metrical Change*,” corresponds to a participant changing his movement tempo from one metrical level to another. That is, the participant divides his movement tempo by a factor of two in a few segments to come closer to a lower metrical level. The fifth pattern, “*Limit Reached*” corresponds to a participant reaching the highest tempo for the session and immediately decelerating afterward. The sixth pattern, referred to as “*Synchronization*” corresponds to a match between the participant's movement and the musical tempo or an equivalent metrical level within 1 BPM from the musical tempo (as in Styns et al., 2007).

The seventh pattern, “*Stable*” corresponds to a short period of time when the movement tempo barely changed or did not change at all. This period of time is usually not longer than 6 consecutive segments. It was decided that accelerations below 3 BPM between two segments or through the course of several consecutive segments were considered as “*Stable*” patterns. This pattern is different from the next “*No Disruption*” pattern as it lasts for a shorter period of time. The eighth and last pattern, “*No Disruption*” corresponds to an inexistent influence of music on movement. This corresponds to the tempo of movement not changing over time during the whole recording session despite the musical tempo increasing. This category is the only one that excludes other categories as only participants that do not show any disruption during the whole session fall in this category.

**Figure 3:**
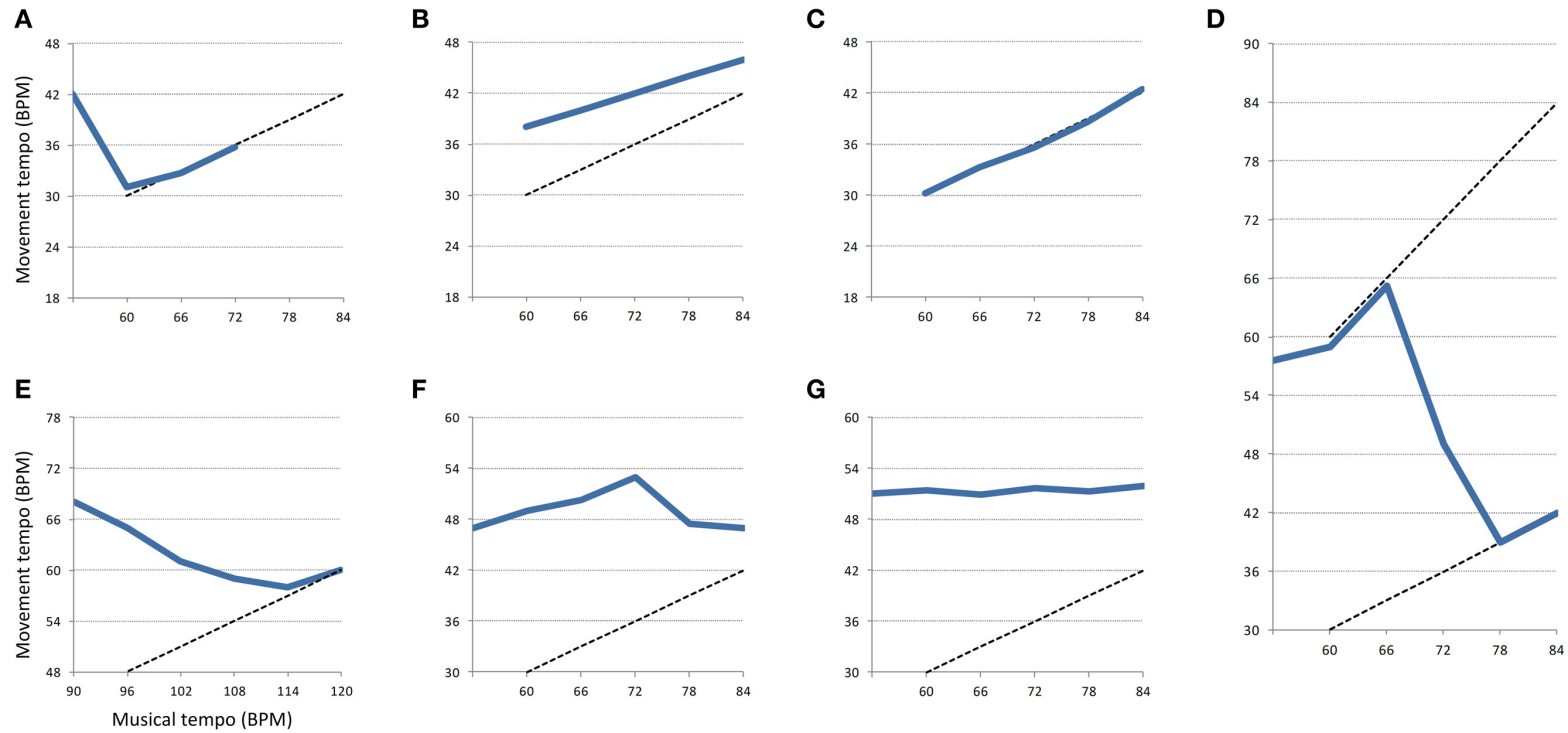
**Examples of behavioral patterns found in the experiment**. Thick blue lines represent the evolution of the movement tempo from segment to segment. Dotted lines represent a perfect match between musical and movement tempo according to a certain metrical level. The represented patterns are the following: **(A)** Start Adaptation, **(B)** Acceleration, **(C)** Synchronization, **(D)** Metrical Change, **(E)** Adaptation, **(F)** Limit Reached, **(G)** Stable. The pattern “No Disruption” is not represented here because it corresponds to a flat line during the whole experiment.

An example of each pattern can be found in Figure 3. Thick blue lines represent the behavior of a hypothetical participant expressing a specific pattern. Dotted lines represent a perfect match between movements and musical tempo according to a certain metrical level.

The number of participants expressing each behavioral pattern can be found in Table 4. Regarding the *Start Adaptation* pattern, the first number in parenthesis corresponds to an acceleration when music starts and the second number to a deceleration when music starts. Note that synchronizations are reported as the number of participants synchronizing at some point and the number in parenthesis corresponds to the total number of synchronization occurrences for a movement. Ultimately, dividing the number of synchronization occurrences by the number of participants gives us the average number of segments during which participants synchronized. It is noteworthy that these segments might not be consecutive as participants can sometimes desynchronize with music before re-synchronizing.

**Table 4:** **Number of participants expressing a specific behavioral pattern during a recording session for each movement**.

**Pattern**	**Movement**			
	**Shoulder**	**Elbow**	**Knee**	**Tapping**
Acceleration	6	10	5	13
Start Adaptation	0	5 (2/3)	0	9 (4/5)
Adaptation	5	3	2	8
Metrical Change	0	1	0	5
Limit Reached	3	5	3	7
Synchronization	10 (11)	13 (22)	9 (12)	13 (52)
Stable	10	9	7	5
No Disruption	4	0	7	0

*Numbers in parenthesis for the Start Adaptation pattern correspond to the number of participants accelerating/decelerating when music started, respectively. Numbers in parenthesis for the Synchronization pattern correspond to the total number of segments during which participants synchronized*.

The different patterns found in participants can be summarized as being indicative of the influence of music on movement or to the contrary, the lack of influence. The first six patterns are synonym of influence while the last two tend to express a lack of influence (see above and below dotted line in Table 4, respectively). Generally speaking, movements tend to show the same organization as in the previous analysis. Namely, the tapping task and the elbow joint movement are the most influenced by music. Shoulder joint movement is influenced to a lesser extent while knee joint movement was hardly influenced by music (seven out of 15 participants were not influenced by the music at all).

Table 4 also gives us information on the number of participants that synchronized with music at some point. As seen above, participants did synchronize with the music on rare occasions. The movement organization follows the same as previously found. Namely, synchronizations were more often observed in the tapping task and in the elbow joint movement.

During the Shoulder movement, participants synchronized on average 4.8% (±6.9) above their baseline (min −9%, max 15%). For the Elbow movement, participants synchronized on average 3.4% (±12.9) below their baseline (min −41%, max 20%). On average, participants synchronized 1.8% (±3.5) above their baseline during the Knee movement (min −6.5%, max 7%). Finally, participants that synchronized in the tapping task did so 14.8% (±32.5) above their baseline (min −25%, max 125%). The case of the Tapping task is particular as many participants synchronized with music during an average of 4 segments; participants increased their tempo significantly in relation to their baseline, leading to higher percentages above their baseline.

To summarize, participants who synchronized did so in a 3–15% zone around their baseline, mostly above it. Synchronizations typically occurred after the *Start Adaptation*, *Adaptation*, and *Metrical Change* patterns, suggesting an attraction of the music on movement. Furthermore, synchronizations also occurred after *Stable* patterns, suggesting that participants were not influenced until their movement tempo was close to the music tempo.

Interestingly, participants behaved differently depending on their baseline tempo when music started. This was observed in the form of Start Adaptation patterns in the Elbow and Tapping tasks. For example, in the Elbow movement, participants that had a Baseline at around 55 BPM and below tended to decelerate toward a 1:2 metrical level (i.e., perceiving one beat every two beats). All the participants above this threshold interacted with music at a 1:1 metrical level at some point in the session. Participants with a Baseline around 75 BPM decelerated toward the 1:1 metrical level when music started. A similar behavior was observed in the tapping task with a threshold situated around the middle of two metrical levels. For example, most participants that moved at around 90 BPM or above synchronized immediately with the 2:1 metrical level when the music started. Participants that moved at a lower tempo during the Baseline interacted with the 1:1 metrical level, mostly expressing *Start Adaptation* and *Synchronization* with music within a few segments. Participants under 60 BPM interacted with the 1:2 metrical level. This would suggest that there is a threshold that determines the metrical level with which participants tend to interact.

#### How did participants subjectively rate different moments in the experiment?

Participants reported music being “too fast” or “too slow” as being unpleasant and difficult when performing oscillatory movements and the tapping task. Furthermore, some participants rated epochs without music (Baseline and Silence) as more pleasant than the rest of the session because of the absence of interferences on their movements. The *Metrical Change* and *Limit Reached* patterns were associated with unpleasant ratings as movements could not keep up with the music. Overall, synchronizations were associated with pleasant ratings.

### Discussion

We investigated how the perception of rhythmic music influenced self-paced movements. Our focus was to unveil the basic mechanisms of entrainment to music through the interaction between motor resonance and motor constraints. In other words, we tried to uncover how motor resonance with music might influence self-paced oscillatory movements using different limbs.

The data we collected are intricate. In order to understand what happened during the experiment, based on four different notable moments during each recording session, we computed three indices: Music Starting (Baseline/Second Segment comparison), Music Stopping (Twelfth Segment/Silence comparison) and Baseline-Silence Difference (Baseline/Silence comparison). These three indices gave us information on how different movements were influenced by the appearance and disappearance of a musical pulse. Overall, we found that all movements were influenced by these events to some extent but the direction of this effect was not consistent (i.e., relative values). This was most likely due to participants behaving in complex and sometimes opposing ways, leading to an absence of a clear effect. The only consistent data point was that participants in the tapping task decelerated when the music stopped by about 10% of their tempo in the last music segment. This deceleration was most striking when participants synchronized with music during the last segment. The sudden absence of the musical pulse most likely led participants to go back to a more comfortable pace. This result is in line with Yu et al. (2003) who found a drift toward the preferred movement tempo when the auditory cue disappeared in a continuation task. When entrainment stopped (i.e., having nothing to resonate to), we could argue that the motor constraints took over and led participants to move closer to their baseline tempo (i.e., resonant frequency).

The tapping task was consistently more affected by the music than other movements. This distinction between the tapping task and other movements is relevant if we consider the different timing mechanisms involved in producing these rhythmical movements. Tapping is an event-based task (Delignières et al., 2004, see Repp, 2005 for a review on tapping) while genuine pendulum-like oscillatory movements are more likely based on spinal CPGs, sending alternating pulses to antagonist muscle groups. Finding greater entrainment in the tapping task suggests that music affected event-based movements to a greater extent than oscillatory movements. We could thus argue that taps were more influenced than oscillatory movements because they rely on the onset of a precise event for the movement to be performed. In the same sense that Naveda and Leman (2010) showed that dance movements were represented in a spatio-temporal format based on the musical beat, taps were more likely to entrain with music due to their event-based nature. This explanation is further supported by the fact that the elbow movement was somewhat more influenced by music than the two more genuine oscillatory movements. Moving the elbow most likely required much more cortical involvement and relied to a lesser extent on CPGs. We could further argue that the other movements were least sensitive to the musical pulse because they do not depend on event-based timing mechanisms. In the absence of a “target” in space and time (e.g., Naveda and Leman, 2010), oscillatory movements were less likely to entrain with music. We could imagine that if participants had performed typical, more ecological dance movements with music, they might have been more influenced by the event-based nature of these dance movements. This particular issue needs to be investigated in further research.

To further understand the strategies put in place by participants to cope with the task, we analyzed the shared behavioral patterns expressed by participants in all movements. The eight patterns described were indicative of either an influence of music on movements or no influence of music on movements. We argue that most of these patterns were performed depending on how the musical tempo was perceived. More precisely, we argue that it was the mismatch between the participant's movement tempo and the perceived musical tempo that led to these patterns. The fundamental assumption would be that participants' movements were attracted to whichever metrical level they were perceiving. The most striking example of this was found in the *Adaptation*, *Start Adaptation*, and *Metrical Change* patterns. Namely, participants either slowed down or accelerated their movements toward a high or lower metrical level (despite the increasing tempo). When music was perceived as faster than their movements, participants were more likely to accelerate. Contrariwise, when it was perceived as being slower than their movements, participants were more likely to slow down. The case of *Metrical Change* is possibly the expression of the motor constraints the participants had to deal with. When the music was too fast for them, they changed their perception of the metrical level and slowed down accordingly. These participants usually synchronized with music after halving their movement frequency, giving further support to the perception account.

As suggested above, the relationship between action and perception can go both ways. Much like Toiviainen et al. (2010) who showed that different parts of the body resonated to different metrical levels, we could argue that participants perceived the music's metrical level depending on the capacity offered by the limb used (i.e., perceptual resonance). This would also be in accordance with Moelants' (2002) suggestion that music perception relies on a perceptual resonance phenomenon. Namely, he established a parallel between the most perceived and represented musical pulse in western dance music (120 BPM) and the frequency at which the motor system seems to be tuned (2 Hz or 120 BM; MacDougall and Moore, 2005). Styns et al. (2007) and Leman et al. (2013) also suggested that walking synchronized with music was the most accurate when the musical tempo was around 120 BPM. In a way, this would also give support to the notion of musical affordances suggesting that music perception relies on motor processes. Furthermore, it would support the idea that motor potentialities in the music should be perceived depending on the capabilities of the motor system. Accordingly, in our experiment, we observed that participants behaved differently depending on their baseline movement tempo when music started. We could thus argue that movements that were performed in silence (before music started) ultimately determined how music was perceived (perceptual resonance), which would have, in turn, determined how participants moved (motor resonance). These observations would thus suggest that participants' resonant frequency might have determined how they perceived the metrical level, leading to their movements adapting to it through motor resonance. In other words, participants might have found in the musical stream the features (i.e., a specific tempo/metrical level) that would allow them to move along to it (i.e., musical affordances). This would be in line with Todd et al. (2007) study that suggested that the perception of musical rhythms might be shaped by the anthropometric factors influencing the natural movement tempo of the individual. Very recently, Dahl et al. (2014) also suggested that music perception or rather preferred beat rate was dictated by body morphology.

Interestingly, participants sometimes were not influenced by music at all. A first explanation could be that participants were focusing exclusively on their movement while paying no attention to the music. This explanation does not hold as we have found these patterns mostly in two movements and not in the others. There must be something else than attentional focus if participants were not able to ignore the music during other specific movements. Therefore, another possibility could be that participants' movements were too slow or too fast to entrain with a specific tempo or equivalent metrical level. In other words, movements were not attracted toward the musical tempo because they were “too far” from it. One way to explain this lack of influence is to consider the specific limbs that represent the *Stable* and *No Disruption* patterns the most: Knee and Shoulder movements. Like previously suggested, their lower sensitivity to background rhythmic music might come from their greater reliance on spinal timing mechanisms meaning they were more finely tuned to an optimal frequency. Furthermore, compared to the two other movements, the lower leg and the whole arm have a far greater inertia. In that sense, it may have required too much effort and energy to deviate from the optimal functioning tempo, going against the “natural” aspect of the required movements. Thus, the motor constraints were probably too strong to overcome. Participants were thus not inclined to accelerate or decelerate their movement tempo in response to music. Furthermore, we could argue that the motor constraints effectively constrained the range within which music was attractive (motor resonance) as synchronization was observed nevertheless during these movements. More precisely, they changed the phase of their movements to be in accordance with the musical pulse when the musical tempo was matching their movement tempo.

Regarding the *Stable* patterns, we could also argue that they were due to the motor constraints not allowing any kind of interaction with music, like it was suggested for Knee and Shoulder movements. However, as they were found in every movement, we cannot exclusively rely on the limb-specific motor constraints explanation. Therefore, we argue that these patterns are a more specific expression of a too great distance between the actual movement and the musical tempo. It is possible that participants managed to inhibit listening to music during these short periods of time but when the musical tempo was closer to the actual movement again, it had an effect on their movements. We could see that as participants “waiting” for the music to become attractive again or maintaining a comfortable tempo as the tempo could not be followed anymore (i.e., the lower metrical level being too far away). At this point, decelerating or accelerating would have required too much energy.

Like Demos et al. (2010), we also found occurrences of synchronization between the participants' movements and the musical tempo. This would suggest that spontaneous entrainment to a background music can occur when attention is not specifically devoted to the music. The fact that participants synchronized is relevant to the assumption that music perception is intrinsically linked to movement. Even more relevant to our motor constraint hypothesis, synchronizations were only found within a 3–15% range around the preferred movement tempo. This zone is in line with what Repp (2006) found in a tapping-continuation task with audio distractors. More precisely, he found that audio distractors were only effective when their tempo was around 10% of the tempo that participants had to reproduce without any auditory cues. Our results further suggested that resonant frequencies are most sensitive to entrainment (as opposed to interference) within a similar range. In our experiment, it was the synchronizations that lasted for extended periods of time in the tapping task that led to this higher range. Musical tempo that was too fast or too slow compared to the movement baseline (resonant frequency) would not lead to synchronization. A particular case can be made of the tapping task as most participants synchronized with the music for extended periods of time. Furthermore, synchronizations even occurred after “*Stable*” patterns, further suggesting that participants were not influenced by music until their movement tempo was close to the musical tempo. In the end, we could argue that participants synchronized with music when it was easy enough or when their movements were close enough to the musical tempo.

Participants were also asked to verbally rate pleasant and unpleasant moments inside each recording session. Moments when participants felt a match between their movements and the music were consistently associated with pleasantness. Moments when participants felt that the music was too slow, too fast were associated with unpleasant ratings. These ratings are in line with the general assumption that “being in the groove” is a pleasant experience (e.g., Janata et al., 2012). Although participants were not instructed to attend the music, one can argue that their ratings suggest that they paid attention to it. Participants were not instructed to do anything special about the music. The fact that participants reported music being “too fast” or “too slow” is indicative that the participants perceived a mismatch between their movements and the music. It can also be indicative that, from a more auditory point of view, participants evaluated fast and slow music as unpleasant to hear. In any case, it would mean that participants could not refrain from attending music and being influenced by it. This further suggests that the spontaneous perception of music (as opposed to listening to it) interacts with ongoing movements. We note that our participants were not specifically instructed to suppress hearing the music. We might imagine that they processed and listened to it, even if they did not explicitly use the music to influence their movements, in line with our instructions.

## General discussion

Our results suggest that music influences movements differently depending on the limb used. The only distinction we found between the different limbs was that the most influenced rhythmic movement was consistently the tapping task and to a lesser extent the elbow movement. The two other movements were almost never influenced by music. We argue that the information extracted from hearing music in the background (the beat) is similar in nature to event-based motor information (taps); the musical pulse is processed in a motoric fashion. This is in line with neuroimagery studies that suggest that rhythms and beats are processed in the motor system (e.g., Grahn and Brett, 2007; Stupacher et al., 2013). This is also in line with Naveda and Leman's (2010) work on dancers. They showed that synchronizing with music involved event-based movements that were based on periodicities of two beats, suggesting that movements were performed according to goals defined in space and time. In our experiment, the participants' task was not to synchronize with music and we still found most synchronization in the most event-based movement. In that sense, it might have been more attractive for participants to synchronize with music due to the nature of the tapping task.

In line with the recent embodied music cognition literature, our study gives support to the notion that music perception is processed in a motoric fashion. As such, it can interfere with ongoing movement but mostly with movements that involve event-based timing mechanisms. To our knowledge, this study is the first to examine spontaneous entrainment to music in such detail. The present research provides experimental data on how music can afford movement depending on motor capabilities of different limbs. In line with Gibson's view on affordances (Gibson, 1979) and the literature on musical affordances (e.g., Godøy et al., 2009, 2010), we tended to show that movements were tuned to, or at least influenced by, the perception of music. We further suggested that the resonant frequency of limbs was an important factor to consider if we want to understand entrainment to music. In that sense, in line with the literature on the concept of groove, we suggest that a match between musical features and motor capabilities is associated with pleasant feelings and a tendency to be influenced by music to a greater extent.

Limitations of the present study might consist of movements not being ecological enough to relate to dance movements and music. Finger tapping is one of the most common ways through which people interact with music and might be used to spontaneously embody the musical pulse (Su and Pöppel, 2012). The three other movements that were chosen were relevant to our biomechanical constraints hypothesis but might not have been the most appropriate to investigate the impact of music. In that sense, we could assume that asking participants to perform typical rhythmic dance movements (e.g., Naveda and Leman, 2010; Toiviainen et al., 2010) rather than genuine pendular oscillatory movements would have led to different results. Namely, it is very likely that we would have observed more synchronization and entrainment to music with more realistic movements. This particular issue should be studied in further research. Another limitation of the study might have been the choice of the musical material. Although they were created with a control on tempo in mind, the different instrumental loops in each song (reflecting different genres) might have had an unsuspected specific effect on movements. As Burger et al. (2013) and Leman et al. (2013) have suggested, certain musical features related to timbre and rhythms (e.g., pulse clarity) have a specific impact on music-induced movements. Moreover, studies on the musical groove have also looked into the musical features that would induce an urge to move along to music (e.g., Madison et al., 2011) and found that beat salience as well as the metrical density were critical.

Future research should investigate the individual factors involved in the different behaviors observed. Namely, it would be of great interest to investigate why certain participants were more sensitive to musical rhythms while others were not. Investigating their dance habits and musical experience might give us insights as to why some participants were more influenced by music than others. Future work on the influence of music on movement should also examine the notion of groove which suggests a close match between motor capabilities and its implication on music-induced movements. In addition, future research should also look into the implications of this tendency to move on music in rehabilitations for example. A better understanding of how music and particularly its rhythmical features can evoke movement seems essential.

### Conflict of interest statement

The authors declare that the research was conducted in the absence of any commercial or financial relationships that could be construed as a potential conflict of interest.

## Footnotes

^1^The purpose of this test was to focus the participant's attention on a different task than the movements they would have done and/or have yet to perform. This was done to avoid the potential influence of a movement (fast or slow) onto the next one; known as the carry-over effect.

^2^The tracks are available online at http://leadserv.u-bourgogne.fr/en/members/mathieu-peckel/pages/extraits

^3^A one-dimensional order-7 median filter works by taking the median value of 7 consecutive data points around one point. Every window of 7 data points is processed separately and the signal is then reconstructed based on the median values. The term one-dimensional refers to the fact that the window is processed on one dimension (the signal amplitude) as opposed to two dimensions in an image or a two-dimensional curve.

^4^While BPM (i.e., beats per minute) is the measuring unit of the musical tempo, movements' frequencies are here computed and referred to as “tempo” for convenience purposes.

## Appendix

Composition of the six songs using Garage Band.

**Table:**

**Stimulus 1**	Funky pop drum 08	**Stimulus 2**	Clave 03
	Straight up beat 02		Shaker 19
	Classic rock steel 03		80s pop beat 07
	80s pop beat 07		Deep house dance beat 04
	Clave 03		80S dance beat synth 03
	Southern rock piano 05		
**Stimulus 3**	Deep house dance beat 06	**Stimulus 4**	Deep house dance beat 01
	Spacey electric piano 01		Upbeat electric piano 03
	Synth tone bass 03		80s pop beat 10
	Funky pop drum 01		Funky pop drum 08
**Stimulus 5**	Upbeat funk drums 03	**Stimulus 6**	Upbeat drums 04
	Latin lounge piano 01		Shaker 16
	Shaker 19		Round latin bass 05
	Woody latin bass 08		Hip-hop beat 02
	Clave 03		Hip-hop beat 01
	Straight upbeat 02		Upbeat funk drums 01

Table of the criteria used to determine the different patterns.

**Table:**

**Pattern**	**Acceleration**	**Deceleration**	**No changes**	**Starting point**	**Number of segments**	**Target**
Acceleration	≥3 BPM	No	No	Anywhere	≥2	No
Start adaptation	Yes	Yes	No	Baseline	Between 2 and 5	Lower or higher metrical level
Adaptation	No	Yes	No	Anywhere	Between 2 and 5	Lower metrical level
Metrical change	No	Yes	No	Metrical level	Between 2 and 5	Lower metrical level
Limit reached	Yes	Yes	No	Anywhere	3	No
Synchronization	Yes	No	No	Anywhere	Between 1 and 11	Metrical level
Stable	No	No	Yes	Anywhere	≤6	No
No disruption	No	No	Yes	Baseline	13	No
